# Protective Effects of Yiqi Xingnao Oral Liquid on Cerebral Ischemia-Reperfusion Injury in Rats and Its Related Mechanisms

**DOI:** 10.1155/2020/3268047

**Published:** 2020-08-25

**Authors:** Tao Pang, Jing Zhao, Feng Zhang, Shujuan Piao, Hong Yang, Jianpeng Jiao, Lei Tang, Wenquan Lu, Wansheng Chen

**Affiliations:** ^1^Department of Pharmacy, Changzheng Hospital, Navy Medical University (Second Military Medical University), Shanghai 200003, China; ^2^Department of Traditional Chinese Medicine, Changzheng Hospital, Navy Medical University (Second Military Medical University), Shanghai 200003, China

## Abstract

**Purpose:**

This study aimed to investigate the effects of different concentrations of Yiqi Xingnao (YQXN) oral liquid on cerebral ischemia/reperfusion (I/R) injury in rats and YQXN's related mechanisms.

**Methods:**

Rats were pretreated with 3 mL/kg, 6 mL/kg, and 12 mL/kg YQXN and Naoxuekang capsule (NXK). Afterwards, cerebral I/R model rats were established by a middle cerebral artery occlusion surgery. Neurological deficits, histopathology, and cerebral infarction volume were used to evaluate the effects of YQXN. Evans blue and terminal deoxynucleotidyl transferase-mediated dUTP nick end labeling (TUNEL) staining were utilized to determine the blood-brain barrier permeability and cell apoptosis, respectively. The expression of VEGF and Bcl-2 was analyzed by real-time quantification PCR (RT-qPCR) and western blot. The malondialdehyde (MDA) content and superoxide dismutase (SOD) activity were measured using corresponding assay kits.

**Results:**

The rats pretreated with YQXN had improved neurological deficits, reduced infarct volume and blood-brain barrier permeability, and ameliorated ischemia-induced morphology change in injured brain tissues. TUNEL staining results showed that different concentrations of YQXN inhibited cell apoptosis of neurocytes in I/R rats. Besides, RT-qPCR and western blot analyses indicated that the expression levels of VEGF and Bcl-2 were significantly upregulated by YQXN compared with the I/R group (*P* < 0.05). Additionally, rats in the I/R group had lower SOD activity and higher MDA content than those in the sham-operated group, while their levels were recovered by YQXN (*P* < 0.05).

**Conclusion:**

YQXN could alleviate cerebral I/R injury by suppressing blood-brain barrier permeability, neuron apoptosis, and oxidative stress, promoting angiogenesis.

## 1. Introduction

Cerebral stroke is a brain tissue injury caused by sudden rupture or blockage of blood vessels in the brain, including ischemic and haemorrhagic stroke [1]. The prevalence of ischemic stroke accounts for approximately 70% of all stroke patients [2], and ischemia/reperfusion (I/R) injury is a major contributor to ischemic stroke, seriously affecting people's health and life quality [3]. Currently, thrombolysis, stent implantation, and drug therapy are the main methods for treatment of ischemic stroke [4]. Due to the short therapeutic window, unstable safety, and significant side effects of long-term drug use, they have major limitations in clinical treatment. Additionally, although tissue plasminogen activator (tPA) is considered the gold standard treatment for ischemic stroke, one of its side effects is enhancement of inflammatory response in brain capillaries and subsequently neuronal cell damage after stroke [5, 6]. Therefore, it is essential to explore the pathological mechanism of cerebral I/R injury and search for effective therapeutic agents with minimal side effects.

It has been reported that the pathological mechanisms of cerebral I/R injury are rather complex and multifactorial, including cell apoptosis, damage by free radicals, oxidative stress, inflammatory response, biological energy depletion, and increased blood-brain barrier permeability [4, 7]. Cell apoptosis is the primary molecular biological mechanism underlying I/R injury and involves the members of the B-cell lymphoma-2 (Bcl-2) family, for example, Bcl-2 (an antiapoptotic protein) [8]. Increasing evidence has shown that oxidative stress and endogenous antioxidant systems are also closely associated with cerebral I/R injury [9, 10]. Additionally, the main functions of the blood-brain barrier are to maintain the microenvironment of brain tissues and transmit central nervous signals [11]. Studies have indicated that blood-brain barrier disruption is recognized as a hallmark of cerebral I/R injury [12].

Traditional medicines are typically composed of natural products which are promising sources of new ingredients for development of conventional medicines. Recent studies have demonstrated that *Scutellaria baicalensis* stem-leaf [13], *Radix Astragali*, *Radix Codonopsis* [14], and other herbal prescriptions [15] exert a positive protective effect on neurological disorders caused by cerebral infarction, indicating that Chinese herbs have become a focus of research for treatment of I/R injury. Yiqi Xingnao (YQXN) oral liquid is improved from Buyang Huanwu decoction, which is a classic prescription for the treatment of stroke. A study by Dou et al. showed that Buyang Huanwu decoction protected against ischemic brain injury by inhibiting infiltration of natural killer cells [16]. However, the effects of YQXN on cerebral I/R injury remain unclear.

Therefore, a cerebral I/R injury rat model was established and pretreated with different concentrations of YQXN. This study aimed to investigate the effects of different concentrations of YQXN on cerebral I/R injury in rats and YQXN's underlying mechanism. These results could provide new clinical therapeutic strategies for cerebral I/R injury and improve our understanding of the occurrence and development of ischemic stroke.

## 2. Materials and Methods

### 2.1. YQXN Preparation

YQXN was made with eleven Chinese herbal medicines. The preparation method of YQXN is as follows: 600 g *Astragalus membranaceus*, 400 g *Ligusticum wallichii*, 200 g *Carthamus tinctorius*, 400 g peach kernel, 400 g *Salvia miltiorrhiza*, 400 g *Concha margaritiferallsta*, 200 g *Arisaema cum bile*, 200 g immature bitter orange, 200 g main polygala, 200 g *Rhizoma acori graminei* and 600 g *Radix Rehmanniae recen* were added to 45.6 L of water. The mixture was then decocted three times, 1 h for each time. After being concentrated, the mixture was alcoholised with 60% ethyl alcohol for 48 h, and then ethyl alcohol was recycled. Thereafter, 100 g sucrose was added to the solution. Finally, after adding 1000 mL water, the YQXN oral liquid was obtained.

### 2.2. Construction of the Cerebral I/R Injury Rat Model and Treatment

A total of 90 SPF male Sprague Dawley (SD) rats weighing 140–160 g were purchased from Shanghai Sipur-Bikai Experimental Animal Co., Ltd. (Shanghai, China). All rats were housed at 22°C–26°C on a 12 h light/dark cycle. During the experiment, the rats were fed with food and water freely. After acclimatization for three days, the rats were randomly and equally divided into six groups (*n* = 15): sham-operated, I/R, I/R + low YQXN, I/R + moderate YQXN, I/R + high YQXN, and I/R + NXK groups. Among them, the rats in the sham-operated and IR groups were gavaged with saline (12 mL/kg). The rats in I/R + low YQXN, I/R + moderate YQXN, and I/R + high YQXN groups were gavaged with 3 mL/kg YQXN, 6 mL/kg YQXN, and 12 mL/kg YQXN, respectively. Besides, the rats in the I/R + NXK group were gavaged with Naoxuekang capsule (0.09 g/kg, NXK). All rats were gavaged once a day for seven days.

One hour after administration on the last day, all rats were anaesthetized with 2% pentobarbital sodium (75 mg/kg), and then a middle cerebral artery occlusion (MCAO) surgery was performed on the rats in the I/R, I/R + low YQXN, I/R + moderate YQXN, I/R + high YQXN, and I/R + NXK groups using line embolism, according to the protocol of Wen et al. [17]. In brief, after anesthesia, the rats' right common carotid artery (CCA) was exposed through a midline neck incision and was distally ligated. The external and internal carotid arteries were then isolated, and MCAO sutures were used for I/R modelling. Two hours after ischemia induction, reperfusion was established by removing the suture. The rats in the sham-operated group underwent the same surgical procedure without occlusion of the CCA. All animal experiments were approved by Animal Experimental Ethical Inspection of Laboratory Animal Centre, Naval Medical University, Changzheng Hospital.

### 2.3. Evaluation of Neurological Deficits

Neurological deficit scores of the rats were evaluated at 24 h after the induction of MCAO by a blinded investigator, as described previously [4]. Neurological deficit scores were graded on a scale of 0–4 as follows: 0, no neurological impairment and normal physical activity; 1, inability to extend the forepaw fully; 2, circling to the opposite side when walking; 3, falling towards the opposite side; and 4, inability to walk spontaneously or loss of consciousness.

### 2.4. Tissue Collection and Histopathology Analysis

The rats were sacrificed after being anaesthetized, and the brain tissues were collected. One part of the brain tissues was fixed with 4% paraformaldehyde (China National Pharmaceutical Group Corporation, Shanghai, China) for histopathology examination. The other part was washed in sterile phosphate buffer saline (PBS), frozen in liquid nitrogen, and stored at −80°C for subsequent studies.

The method of histopathology analysis was described previously [18]. In brief, the brain tissues were fixed in 4% paraformaldehyde and embedded in paraffin. The 5 *μ*m sections were cut and stained with haematoxylin-eosin (HE). Slides were scanned, and images were taken under optical microscopy (Olympus Corporation, Tokyo, Japan). The histopathological scoring criteria are shown in Table 1 [19].

### 2.5. Determination of the Cerebral Infarction Area

The brain tissues were sectioned into 2 mm sections and fixed in 4% paraformaldehyde for 4 h. Afterwards, these sections were stained with 2,3,5-triphenyltetrazolium chloride (TTC, Bio Basic Inc., Shanghai, China) according to the manufacturer's instructions. After TTC staining, the slide images were photographed, and the cerebral infarction volume was calculated using ImageJ 1.6.0 (National Institutes of Health, USA) according to the following formula: the percentage of infarct volume (%) = area of infarcted tissue/area of the whole brain × 100%.

### 2.6. Blood-Brain Barrier Permeability Analysis

After reperfusion of 24 h, five rats in each group were injected with 2% (w/v) Evans blue (EB, 4 mL/kg, China National Pharmaceutical Group Corporation, Shanghai, China) through the tail vein. After color of the rats' lips, bulbar conjunctiva, and limbs had turned blue for two hours, all rats were anaesthetized with 2% pentobarbital sodium (75 mg/kg) and perfused with 200 mL saline to eliminate the remaining EB. Afterwards, the injured brain tissues (100 mg) were incubated with formamide (3 mL) at 50°C for 72 h and centrifuged at 1500 r/min for ten minutes. The absorbance value of the supernatant was measured using a microplate reader (Bio-Rad Laboratories, Hercules, CA, USA) at 630 nm. An EB standard curve was drawn as previously described [13], and the EB content (*μ*g/g) was then calculated based on the standard curve.

### 2.7. Cell Apoptosis Analysis

The terminal deoxynucleotidyl transferase-mediated dUTP nick end labeling (TUNEL) method was used to determine cell apoptosis of neurocytes in brain tissues. The brain tissues (50 mg) of rats in different groups were fixed in 4% paraformaldehyde for 30 min and washed with PBS. After being incubated at 4°C for two minutes, the samples were dehydrated with decreasing percentages of ethanol (100%, 95%, 90%, 80%, and 70%) and then washed with PBS three times. Afterwards, the brain samples were incubated with 50 *μ*L TUNEL reagent (Beyotime Biotechnology Co., Ltd., Shanghai, China) at 37°C in the dark for 60 min. After washing with PBS, 0.2 mL reaction stop buffer was added, and the mixture was incubated at 20°C for ten minutes. Then, 50 *μ*L working liquid was added and then incubated at 20°C in the dark for 30 min. Thereafter, the samples were redyed with haematoxylin for five minutes. After being washed with PBS three times (five minutes each time), the cell apoptosis of the neurocytes was observed under a 400× fluorescence microscope.

### 2.8. Western Blot Analysis

The total proteins of rats in different groups were extracted using RIPA lysis buffer (Beyotime Biotechnology Co., Ltd.), and the protein concentrations were measured by a BCA Protein Assay Kit (Beyotime Biotechnology Co., Ltd.) following the manufacturer's instructions. Subsequently, an equivalent amount of protein (20 *μ*g/lane) from each sample was separated by SDS-PAGE and transferred to PVDF membranes. After blocking with 5% nonfat milk at 37°C for 1 hour, the membranes were incubated with the anti-VEGF antibody (Abcam, 1 : 1000, Cambridge, UK), anti-Bcl-2 antibody (Abcam, 1 : 1000), and anti-GAPDH antibody (Abcam, 1 : 1000) at 4°C overnight. After washing with Tris-buffered saline containing 0.1% Tween 20 three times, the membranes were incubated with goat anti-mouse IgG-HRP (1 : 2000, Jackson ImmunoResearch Laboratories, Inc., West Grove, PA, USA) at 37°C for two hours. After washing, the protein bands were visualized with an enhanced chemiluminescence detection kit (Santa Cruz Biotechnology Inc., CA, USA) and quantified by protein grey-scale analysis using ImageJ 1.6.0.

### 2.9. Real-Time Quantitative PCR (RT-qPCR)

Total RNA was isolated from brain tissues in different groups using TRIzol reagent (Thermo Fisher Scientific (China) Co., Ltd., Shanghai, China) according to the manufacturer's instructions. The RNA concentration and quality were assayed by a microplate reader. Thereafter, the isolated RNA was reversely transcribed into cDNA using PrimeScript™ II 1st Strand cDNA Synthesis Kit (Beyotime Biotechnology Co., Ltd.) based on the manufacturer's instructions. All primers were designed and synthesized by Sangon Biotech Co., Ltd. (Shanghai, China), and the sequences of all primers are shown in Table 2. The total reaction volume was 10 *μ*L, including 5 *μ*L PCR Master Mix, 0.5 *μ*L forward primer (10 *μ*M/L), 0.5 *μ*L reverse primer (10 *μ*M/L), 1 *μ*L Cdna, and 3 *μ*L diethyl pyrocarbonate water. The reaction conditions were displayed as the following: 95°C for ten minutes, 95°C for 15 s, and 60°C for 60 s for a total of 40 cycles. The relative mRNA expression levels of vascular endothelial growth factor (*VEGF*) and *Bcl-2* were calculated using the 2^−ΔΔCt^ method [20]. All values were normalized to the reference gene GAPDH, and data were obtained from three independent experiments performed in triplicate.

### 2.10. Determination of Biochemical Indices

To evaluate the extent of oxidative stress in rats treated with I/R and YQXN, the malondialdehyde (MDA) content and superoxide dismutase (SOD) activity were determined using assay kits for MDA and total SOD from Beyotime Biotechnology Co., Ltd. (Shanghai, China), respectively. The experiments were performed according to the manufacturer's instructions.

### 2.11. Statistical Analysis

Data were reported as mean ± standard deviation (SD). Graphpad prism 5 (Graphpad Software, San Diego, CA) was utilized to carried out all the statistical analyses. *P* < 0.05 was considered statistically significant.

## 3. Results

### 3.1. The Effects of YQXN on Neurological Deficits and Cerebral Infarction Area in Cerebral I/R Rats

**Figure 1:**
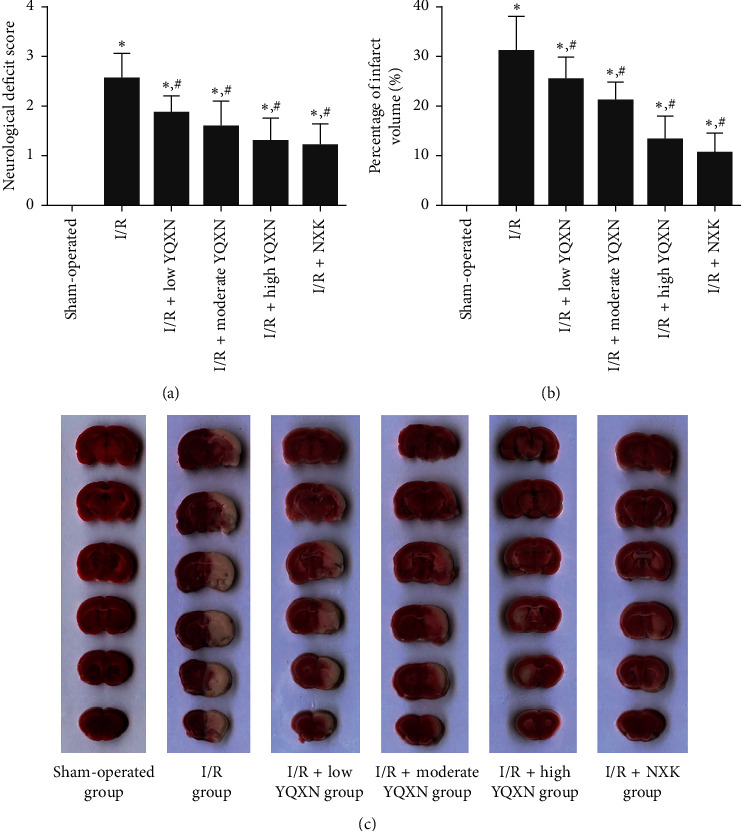
Inhibitory effects of Yiqi Xingnao (YQXN) oral liquid on neurological deficits and infarct volume after ischemia/reperfusion (I/R). (a) The neurological deficit scores. (b) 2,3,5-Triphenyltetrazolium chloride (TTC) staining (white) indicating infarct sizes in rats with different treatments. (c) Percent of infarct volume based on the TTC staining results. ^*∗*^*P* < 0.05, compared with the sham-operated group; ^#^*P* < 0.05, compared with the I/R group.

Neurological deficit and TTC staining are the terminal indicators for the evaluation of cerebral ischemia injury. In the sham-operated group, no rats had neurological deficits (Figure 1(a)). Neurological deficit scores were significantly increased after cerebral I/R injury (I/R group, *P* < 0.05). YQXN pretreatment at different doses evidently attenuated the neurological deficits of rats compared with the I/R group (*P* < 0.05). Additionally, the rats in the I/R + high YQXN group and I/R + NXK group had lower neurological deficit scores than those in the I/R + low YQXN group (*P* < 0.05), and there was no statistically significant difference between the I/R + high YQXN group and the I/R + NXK group (*P* > 0.05, Figure 1(a)).

The changing trend of the cerebral infarction area was similar with that of neurological deficit scores (Figures 1(b) and 1(c)). The cerebral infarction area of rats in the I/R group was significantly increased (*P* < 0.05) compared with the sham-operated group. In contrast, the infarction area was inhibited by YQXN with different doses and NXK (*P* < 0.05, Figure 1(c)). Besides, the cerebral infarction area of rats in the I/R + high YQXN group was close to that in the I/R + NXK group (*P* > 0.05). Taken together, the high-dose YQXN and NXK had similar protective effects on neurological deficits and cerebral infarction area in cerebral I/R rats.

### 3.2. Histopathological Analysis

**Figure 2:**
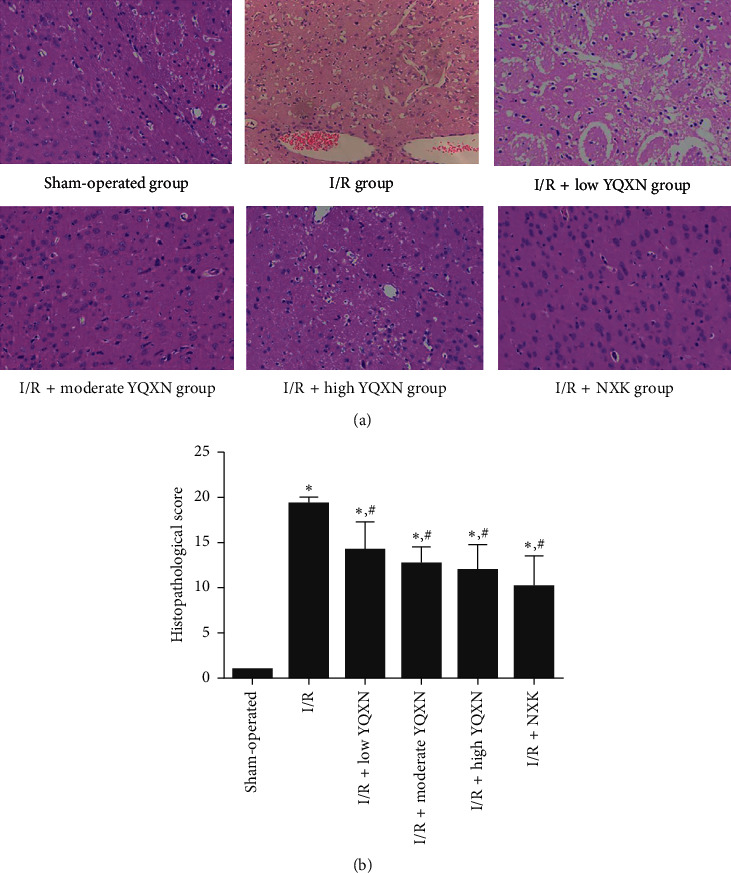
Histopathological change of the brain injured tissue in I/R rats with different treatments. (a) Images of HE staining. (b) Histopathological scores of rats in different groups. ^*∗*^*P* < 0.05, compared with the sham-operated group; ^#^*P* < 0.05, compared with the I/R group.

To further investigate the effects of YQXN on cerebral I/R injury, HE staining was performed on the brain tissues of rats from different groups. In the sham-operated group, the rats showed normal brain neuronal arrangement, normal neurocytes, and no infiltration of inflammatory cytokines. However, the rats in the I/R group displayed disordered brain neuronal arrangement, rupture and swelling of neurons, and obvious infiltration of inflammatory cytokines. The symptoms of cerebral I/R injury in the I/R + low YQXN, I/R + moderate YQXN, I/R + high YQXN, and I/R + NXK groups were alleviated to varying degrees (Figure 2(a)). Furthermore, the histopathological score of rats in the I/R group (19.38 ± 0.74) was significantly higher than that in the sham-operated group (1 ± 0.12, *P* < 0.05). After being pretreated with YQXN and NXK, the histopathological scores were evidently decreased compared with the I/R group (*P* < 0.05, Figure 2(b)).

### 3.3. The Effects of YQXN on Blood-Brain Barrier Permeability in Cerebral I/R Rats

**Figure 3:**
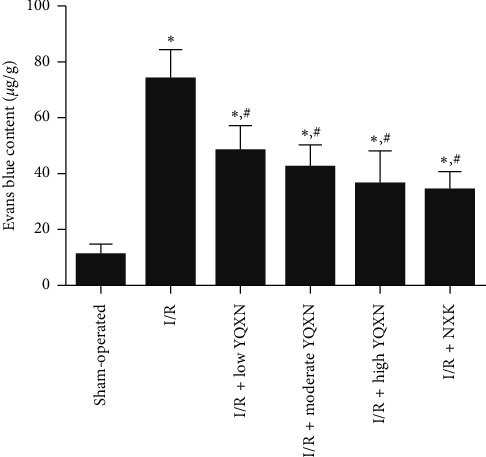
Effect of different concentrations of YQXN pretreatment on Evans blue content in I/R rats. ^*∗*^*P* < 0.05, compared with the sham-operated group; ^#^*P* < 0.05, compared with the I/R group.

The EB content in the sham-operated group was 10.93 ± 4.16 *μ*g/g, while the EB content in the I/R group rose to 74.1 ± 10.57 *μ*g/g, which indicated that cerebral I/R injury could lead to a significant increase in blood-brain barrier permeability (*P* < 0.05). After treatment with YQXN and NXK, the EB content was significantly decreased (*P* < 0.05), and the high-dose YQNX had a similar effect on the blood-brain barrier with NXK (Figure 3). The results indicated that YQXN could alleviate the permeability of the blood-brain barrier induced by I/R, and the effect of high-dose YQXN was better.

### 3.4. The Effects of YQXN on Cell Apoptosis of Neurocytes in Cerebral I/R Rats

**Figure 4:**
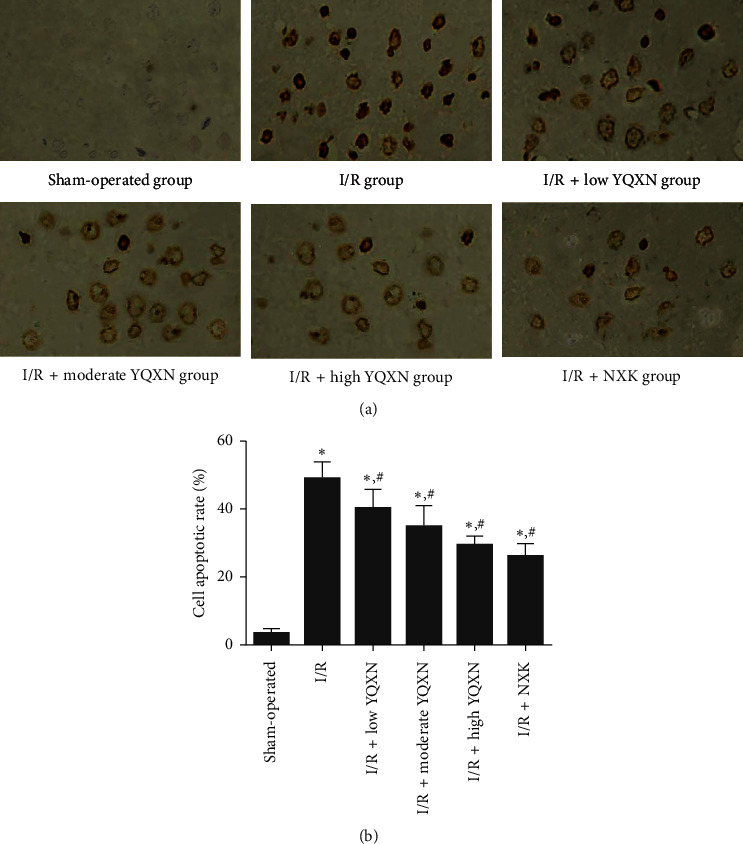
Effect of different concentrations of YQXN pretreatment on cell apoptosis in I/R rats. (a) The images of TUNEL staining. (b) The cell apoptotic rate of neurocytes in different groups. ^*∗*^*P* < 0.05, compared with the sham-operated group; ^#^*P* < 0.05, compared with the I/R group.

TUNEL staining was used to evaluate the effects of YQXN on cell apoptosis of neurocytes in cerebral I/R rats. The representative images of TUNEL staining are shown in Figure 4(a). The cell apoptotic rate in the I/R group was significantly increased compared with the sham-operated group (*P* < 0.05), suggesting that I/R injury could promote the apoptosis of neurocytes in brain tissues. Additionally, the cell apoptotic rates in the I/R + low YQXN, I/R + moderate YQXN, and I/R + high YQXN groups were evidently lower than those in the I/R group (*P* < 0.05), and the apoptotic rate in the I/R + high YQXN group was close to that in the I/R + NXK group (*P* > 0.05, Figure 4(b)). Taken together, YQXN may alleviate the cerebral I/R injury by inhibiting cell apoptosis of neurocytes.

### 3.5. The Effects of YQXN on the Expression Levels of VEGF and Bcl-2

**Figure 5:**
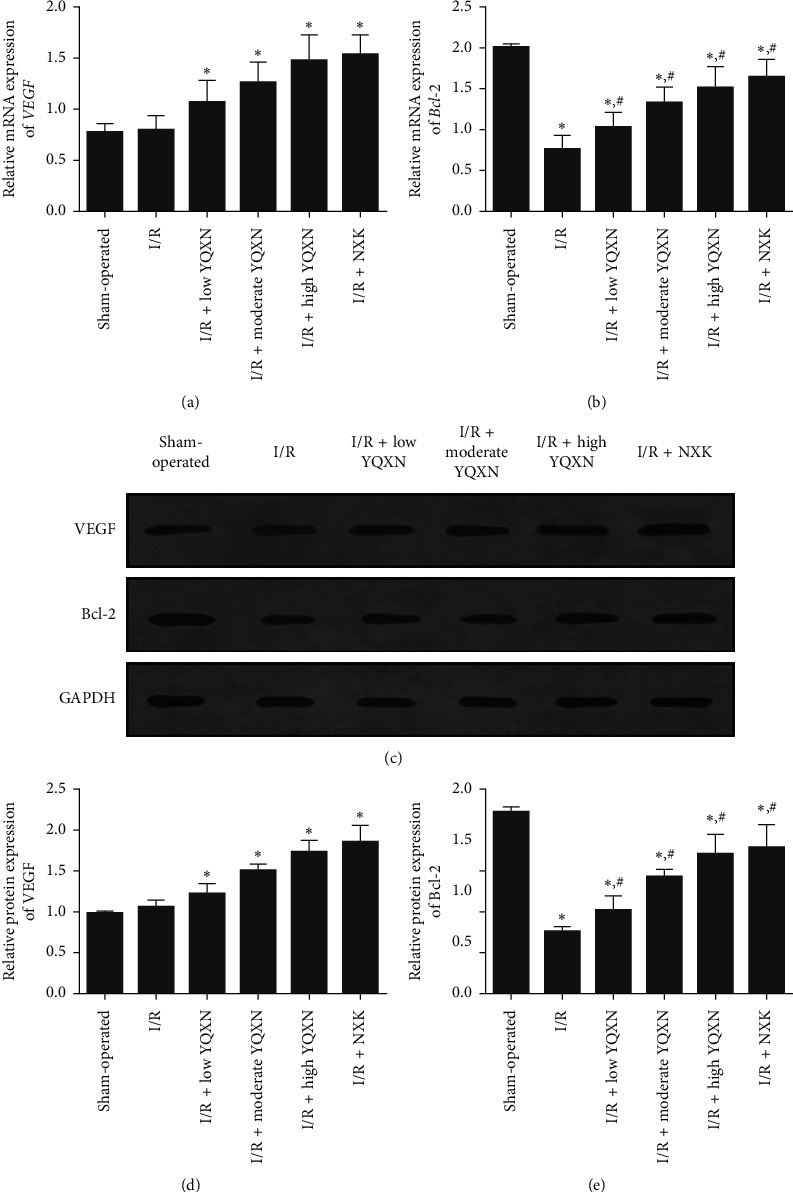
The expression levels of VEGF and Bcl-2 in brain tissues of different groups. (a) The relative mRNA expression level of *VEGF* determined by real-time quantitative PCR (RT-qPCR). (b) The relative mRNA expression level of *Bcl-2* determined by RT-qPCR. (c) The protein expression levels of VEGF and Bcl-2 determined by western blot. (d) The grey analysis of VEGF protein. (e) The grey analysis of Bcl-2 protein. ^*∗*^*P* < 0.05, compared with the sham-operated group; ^#^*P* < 0.05, compared with the I/R group.

The expression levels of VEGF and Bcl-2 were analyzed using RT-qPCR and western blot. RT-qPCR results showed that there was no significant difference in the mRNA expression level of *VEGF* between the sham-operated group and the I/R group (*P* > 0.05), while its expression level increased with the increased concentration of YQXN (Figure 5(a)). Furthermore, the mRNA expression level of *Bcl-2* in the I/R group was lower than that in the sham-operated group (*P* < 0.05, Figure 5(b)). After administration of YQXN at different concentrations, *Bcl-2* expression level was gradually increased, and its expression level in the I/R + high YQXN group was similar with that in the I/R + NXK group (*P* > 0.05, Figure 5(b)). In addition, the changing trend of the expression levels of VEGF and Bcl-2 detected by western blot analysis was consistent with the results obtained by RT-qPCR (Figures 5(c) and 5(e)).

### 3.6. The Effects of YQXN on SOD Activity and MDA Content

**Figure 6:**
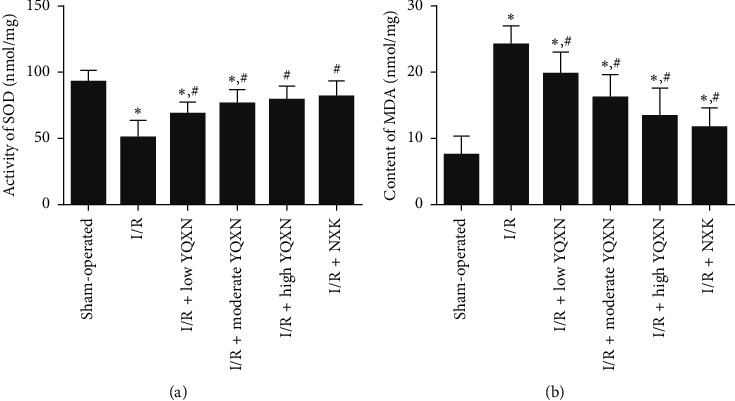
Effect of different concentrations of YQXN pretreatment on (a) superoxide dismutase (SOD) activity and (b) MDA content in I/R rats. ^*∗*^*P* < 0.05, compared with the sham-operated group; ^#^*P* < 0.05, compared with the I/R group.

The activity of SOD in brain tissues of the I/R group was significantly decreased compared with the sham-operated group (*P* < 0.05). After being treated with YQXN, the activities of SOD in brain tissues were all significantly enhanced compared with the I/R group (*P* < 0.05, Figure 6(a)). Besides, the SOD activities in the I/R + high YQXN group and I/R + NXK group were, respectively, 79.69 ± 10.05 nmol/mg and 81.53 ± 11.32 nmol/mg, which were similar with the level in the sham-operated group (92.86 ± 8.87 nmol/mg, *P* > 0.05, Figure 6(a)). However, the change trend of MDA contents in different groups was opposite to that of SOD activity. The MDA content in the I/R group was significantly higher than that in the sham-operated group (*P* < 0.05), whereas the MDA content was evidently inhibited by YQXN and NXK compared with the I/R group (*P* < 0.05, Figure 6(b)).

## 4. Discussion

The heart and the brain are the two most important organs, in which I/R injury plays the most crucial role, causing the highest mortality and morbidity burden to the society [21]. YQXN is composed of 11 kinds of Chinese herbal medicines and has the effects for benefiting Qi for activating blood circulation and restoring consciousness. This study proved for the first time that YQXN exerts a remarkable protective effect on rats with cerebral I/R injury. In the present study, a cerebral I/R model was successfully constructed by MCAO surgery and pretreated with different concentrations of YQXN. We found that high-dose YQXN had better inhibitory effects on neurological deficits and infarct volume of I/R injury rats, which was similar to NXK (*P* > 0.05). EB and TUNEL staining showed that YQXN not only significantly suppressed the increase of blood-brain barrier permeability induced by I/R but also inhibited the cell apoptosis of neurocytes. Additionally, YQXN could alleviate cerebral I/R injury by upregulating the expression levels of VEGF and Bcl-2 and mediating oxidative stress reaction through increasing SOD activity and decreasing MDA content.

Cerebral I/R injury can lead to nerve injury and cerebral infarction [22]. Neurological deficit score, infarct volume, and degree of the lesion are the specific indicators for evaluating brain injury. Furthermore, the blood-brain barrier, consisting of endothelial cells, tight junctions, astrocytic end-feet processes, pericytes, and basement membrane, is essential in regulation of the passage of inflammatory cytokines, proteins, and ions between the brain and plasma [23]. Studies have shown that disruption of the blood-brain barrier in ischemic stroke could increase the permeability and change the microenvironment of the brain, which may contribute to the further development of brain damage [5–12, 24]. Zhang et al. demonstrated that a novel IL-1RA-PEP fusion protein could effectively improve cerebral I/R injury by inhibiting the blood-brain barrier permeability [25]. In our study, NXK was chosen as the positive drug, and we found that YQXN could ameliorate brain injury induced by I/R in a dose-dependent manner. The protective effect of YQXN at 12 mL/kg was similar with that of NXK treatment. Besides, YQXN pretreatment significantly reduced blood-brain barrier permeability in the I/R rats. Combined with our results, it is speculated that high-dose YQXN may have a better protective effect on cerebral I/R injury by lowering the blood-brain barrier permeability.

Increasing evidence has shown that cell apoptosis is involved in cerebral I/R injury [26–28]. In this study, increased cell apoptosis of neurocytes and decreased expression level of Bcl-2 were observed in the I/R rats. However, cell apoptosis was inhibited, and the expression level of Bcl-2 was upregulated when pretreated with YQXN. Bcl-2, an antiapoptotic protein, is an important factor in determining whether apoptosis occurs and the severity of apoptosis [29, 30]. A study by Liu et al. [31] indicated that apigenin could inhibit cell apoptosis by increasing the expression level of Bcl-2, thus protecting renal I/R injury. Another study also showed that *Radix Ilicis Pubescentis* total flavonoids could alleviate cerebral I/R injury via increasing the Bcl-2 protein expression and enhancing the antiapoptotic ability of neurocytes [32]. Taken together, YQXN may play a mitigating role in cerebral I/R injury by upregulating the expression of Bcl-2 and inhibiting the apoptosis of neurocytes.

In addition, VEGF, an effective trigger of angiogenesis, can change the microcirculation and improve survival and regeneration of neurocytes through angiogenesis in ischemic tissues [33]. Increasing evidence has shown that the combination of VEGF and its receptors can trigger multiple downstream signals and promote angiogenesis [34, 35]. A study by Peng et al. [36] indicated that isoflurane increased the expression level of VEGF and enhanced angiogenesis by activating the Shh/Gli signaling pathway, thus ameliorating cerebral I/R injury in rats. In another study, Buyang Huanwu decoction was reported to exert neuroprotection targeting angiogenesis through upregulating expression levels of VEGF and SIRT1 against cerebral ischemic injury in rats [37]. In our study, the expression level of VEGF was significantly elevated by YQXN (*P* < 0.05), while its level in the I/R group was close to that in the sham-operated group (*P* > 0.05). These results demonstrated that YQXN may accelerate angiogenesis in cerebral ischemic tissues via upregulation of VEGF expression, which consequently inhibits the blood-brain barrier permeability and relieves cerebral I/R injury.

Oxidative stress is one of the main mechanisms in I/R injury, including the generation of oxygen free radicals and lipid peroxidation [38, 39]. SOD plays an important role in protecting cells from oxidative damage by converting O^2−^ into H_2_O_2_ and maintaining the balance of oxygen free radicals in the body [40]. MDA is the end production of lipid peroxidation by free radicals, so as to be an indirect indicator of oxygen radicals in tissues [41, 42]. When excess free radicals are generated and accumulated in the body, they can cause neuronal damage. A study reported that compared with the cerebral I/R injury rats, rats treated with Shenqi Fuzheng injection displayed lower MDA content and higher SOD activity, which indicated that Shenqi Fuzheng injection may have the ability to scavenge oxidative radicals [14]. In the present study, rats in the I/R group had lower SOD activity and higher MDA content than those in the sham-operated group. However, their levels were obviously recovered by YQXN. Combined with our results, we speculated that YQXN may mediate oxidative stress by regulating SOD activity and MDA content, thereby attenuating cerebral I/R injury.

## 5. Conclusions

In conclusion, YQXN could exert protective roles in cerebral I/R injury, and high-dose YQXN had a better effect. The underlying mechanisms may be involved in the suppression of blood-brain barrier permeability and neuron apoptosis by upregulating the expression levels of VEGF and Bcl-2. Additionally, YQXN may promote the formation of new blood vessels by upregulating the expression of VEGF and inhibit the oxidative stress reactions by enhancing SOD activity and decreasing MDA content, thus protecting rats from cerebral I/R injury. Our findings will provide new insights into the therapeutic strategy of YQXN for cerebral I/R injury and improve our understanding of the occurrence and development of ischemic stroke.

## Figures and Tables

**Table 1:** Tissue injury scoring criteria.

Items	Severity	Score
Necrotic range (area of brain tissue necrosis/the entire affected side of the brain)	None	0
	1%–25%	1
	26%–50%	2
	51%–75%	3
	76%–100%	4
Degree of the lesion	None	0
	Slight	1
	Little	2
	Moderate	3
	Serious	4
	Extreme serious	5

**Table 2:** The sequences of all primers.

Gene	Primer sequence (5′-3′)
*VEGF*	F: CTGGATATGTTTGACTGCTGTGGA
	R: GTTTCTGGAAGTGAGCCAATGTG
*Bcl-2*	F: TTTCATATTTGTTTGGGCAGGTC
	R: ATGGGGTGAACTGGGGGAGGATTG
*GAPDH*	F: TGTGAACGGATTTGGCCGTA
	R: CATTTGATGTTAGCGGGATC

## Data Availability

The dataset used and/or analyzed during the current study is available from the corresponding author upon reasonable request.
